# A case report of metastatic adenocarcinoma of the gingiva

**DOI:** 10.4103/0972-124X.51898

**Published:** 2009

**Authors:** Aravind Buddula

**Affiliations:** *Department of Periodontics, Dental Specialties, Mayo Clinic, Rochester Minnesota - 559 01, USA*

**Keywords:** Adenocarcinoma, gingival enlargement, metastasis

## Abstract

Localized gingival enlargement is often associated with specific systemic medication, abscess formation, trauma or reactive lesions. Scant literature is available reporting enlargement of gingiva due the metastasis of adenocarcinoma from lung. The case report presents a unique case of an adenocarcinoma in the lung metastasizing to the buccal and lingual interdental papillae of teeth numbering 34 and 35. A 72-year-old female was referred to the Mayo Clinic with a recent diagnosis of metastatic stage IV adenocarcinoma of the left lung presented with an abnormal mass located on the left posterior buccal keratinized tissue adjacent to teeth numbering 34-35. Biopsy of the lesion was performed for CK7, CK20, TTF-1 and p63. The tumor cells were positive for CK7 and TTF-1, and weakly positive for p63 suggesting a diagnosis of adenocarcinoma. The periodontist may be in the unique position to be the first oral health care provider to evaluate any biopsy suspicious intra-oral lesions.

## INTRODUCTION

Malignant metastatic involvement of the oral tissues has been infrequently reported. The overall incidence has been reported to be 1-3% of all oral malignancies.[1,2] Previous publications on metastatic oral malignancies over five decades elucidated that the common sites of metastasis are the breast, lung and kidney.[2,3] A recent review of the 547 metastatic malignancies confirmed that breast, lung, kidney, thyroid and prostate are the most common primary sites.[4]

Furthermore, metastasis to soft tissue is less common compared to the osseous structures.[5–8]

Metastasis to gingival tissues is very rare consequently there are few documented cases of metastatic gingival tumors. Cases of intraoral metastasis have been reported from primary chondrosarcoma of femur,[9] adenocarcinoma of lung,[10] gastric cancer[11] and hypernephroma.[12] This is a case report of a patient with adenocarcinoma of the lung which metastasized to left posterior buccal and lingual gingiva.

## CASE REPORT

A 72-year-old female was referred to the Mayo Clinic with a recent diagnosis of metastatic stage IV adenocarcinoma of the left lung. The patient was a non-smoker, initially visited her primary care physician with a chief complaint of a chronic cough that did not respond to antibiotic therapy. On October 12, 2007, a chest CT scan was performed which demonstrated a left lower lobe mass, as well as a left pleural effusion, and a liver mass. On October 18, 2007, thoracentesis demonstrated evidence of malignant cells. Approximately two liters of fluid was removed. A PET scan performed on October 22, 2007 showed diffuse hypermetabolic uptake in the left upper chest wall, liver, left femur, and left lower lung. A repeat CT scan on October 25, 2007 demonstrated re-accumulation of the pleural fluid. A mass within the right lobe of the liver and lower lung was also evident. The patient was referred to an oncologist.

On November 1, 2007, as a part of the examination, the medical oncologist detected an abnormal mass located on the left posterior buccal keratinized tissue adjacent to tooth number 34, 35. No lymphadenopathy was detected. On the same day, the patient was referred to the Periodontics division, Department of Dental Specialties, Mayo Clinic.

**Figure 1:**
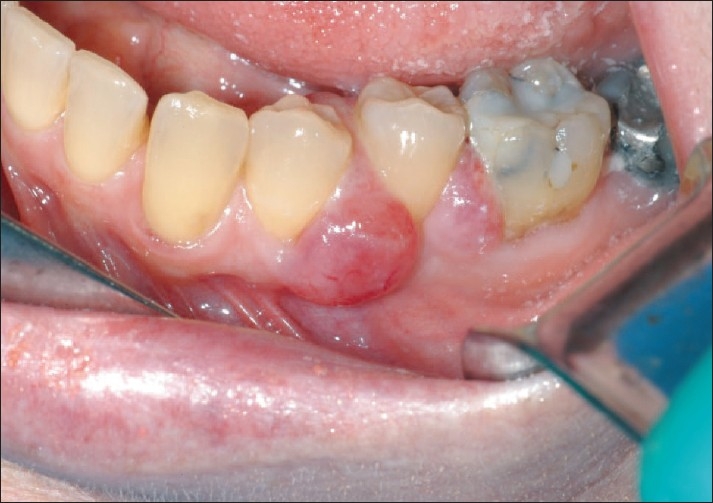
Metastatic adenocarcinoma on the left buccal gingiva in relation to teeth # 34 and 35

**Figure 2:**
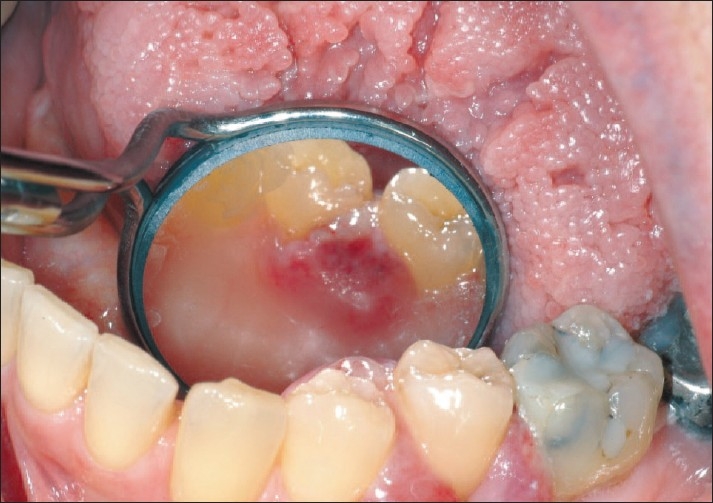
Lingual view of the metastatic adenocarcinoma

An intraoral examination revealed an erythematous growth located on the facial and lingual interdental papillae of the teeth numbering 33 and 34 [Figure 1] and 34 and 35 [Figure 2]. The probing depths throughout the mouth were within normal limits and the patient denied a recent history of trauma. The lesion measured approximately 10 mm in diameter buccal to tooth number 34 and 7 mm in diameter on buccal to tooth number 35. On palpation, the lesion was firm and painless. In addition, lingual papilla interproximal to tooth number 34 and 35 appeared erythematous and edematous. An excisional biopsy was performed on the buccal interdental gingiva of tooth number 34 and 35 and submitted for histologic examination.

### Pathology report

**Figure 3:**
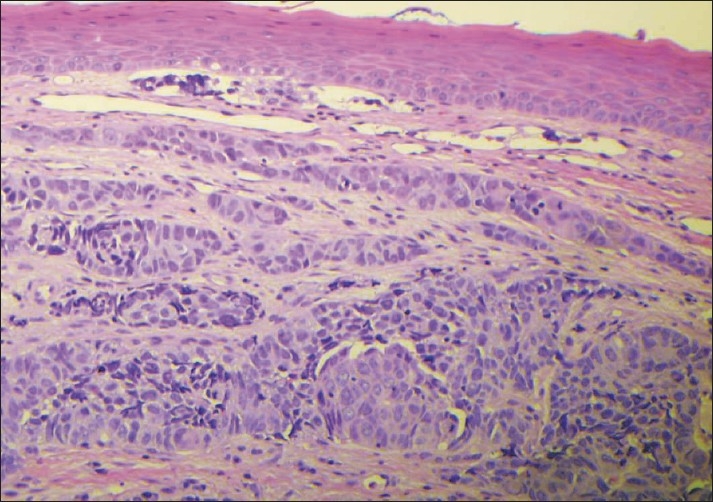
Histologic section of the adenocarcinoma showing squamous mucosa with infiltrating sub mucosal tumor

**Figure 4:**
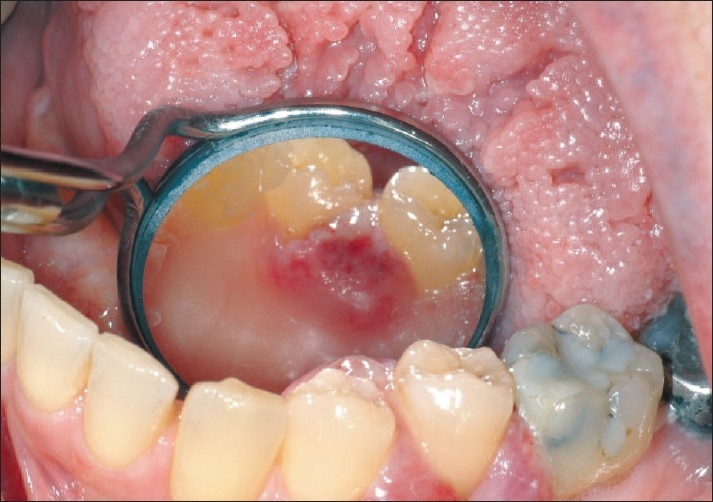
TTF-1 immunostain highlights the tumor cells

Immunohistochemical stains were performed for CK7, CK20, TTF-1 and p63. Histologic section of the adenocarcinoma showed squamous mucosa on top with infiltrating sub mucosal tumor. Some tumor cells nests involve sub mucosal lymphatic spaces. The tumor cells were positive for CK7 and TTF-1, weakly positive for p63 and negative for CK20 [Figures 3 and 4]. These results support the diagnosis of adenocarcinoma.

## DISCUSSION

Metastasis of adenocarcinoma from the lung to oral tissues has been rarely reported.[1] Any suspicious lesion in the oral cavity in a patient with a history of carcinoma should be thoroughly evaluated and biopsied. No studies are available reporting preferential site for metastasis in the oral cavity with regard to adenocarcinoma of lung. Studies involving gastric cancer have reported that gingival metastasis is more common at the anterior region than in the posterior region. This patient came with metastasis in the mandibular left premolar region.[13]

A general dentist or primary care physician may refer suspicious intraoral lesions to the periodontist for further evaluation. The periodontist may be in the unique position to be the first oral health care provider to evaluate and biopsy suspicious intraoral lesions. While rare, primary and metastatic lesions occur approximately 1-3% of the time,[1,2] it is very important that a thorough soft and hard tissue examination be performed as part of an initial periodontal evaluation.

## References

[1] Cash CD, Royer RQ, Dahlin DC (1961). Metastasis tumors of the jaws. Oral Surg Oral Med Oral Pathol.

[2] Meyer I, Shklar G (1965). Malignant tumors to mouth and jaws. Oral Surg Oral Med Oral Pathol.

[3] Clausen F, Poulsen H (1963). Metastatic carcinoma to the jaws. Acta Pathol Microbiol Scand.

[4] Hirshberg A, Buchner A (1995). Metastatic tumors to the oral region: An overview. Eur J Cancer B Oral Oncol Br.

[5] Sactis RL (1964). Metastatic Carcinoma to the jaws bones. Dent Res.

[6] Astacio JN, Alfaro C (1969). Oral mucosa metastasis from gastric adenocarcinoma. Oral Surg Oral Med Oral Pathol.

[7] Lopez N, Lobos N (1976). Metastatic adenocarcinoma of gingiva. J Periodontol.

[8] Abrams HL, Spiro R, Goldstein N (1950). Metastasis in carcinoma: Analysis of 1000 autopsied cases. Cancer.

[9] Hardman FG (1949). Secondary sarcoma preventing clinical appearance of fibrous epulis. Br Dent J.

[10] Humphrey AA, Amos NH (1936). Metastatic gingival adenocarcinoma from primary lesion of colon. Am J Cancer.

[11] Shimoyama S, Seto Y, Aoki F, Ogawa T, Toma T, Endo H (2004). Gastric cancer with metastasis to the gingiva. J Gastroenterol Hepatol.

[12] Willis R, Glickman (1948). Pathology of tumors.

[13] Osaki T, Ryoke K, Hamada T (1978). Metastatic adenocarcinoma to the oral cavity: Report of two cases and review of literature. Jpn J Stomatol Soc.

